# Effect of tauroursodeoxycholic acid on survival and safety in amyotrophic lateral sclerosis: a retrospective population-based cohort study

**DOI:** 10.1016/j.eclinm.2023.102256

**Published:** 2023-10-05

**Authors:** Elisabetta Zucchi, Umberto Maria Musazzi, Guido Fedele, Ilaria Martinelli, Giulia Gianferrari, Cecilia Simonini, Nicola Fini, Andrea Ghezzi, Maria Caputo, Elisabetta Sette, Veria Vacchiano, Lucia Zinno, Pietro Anceschi, Elena Canali, Marco Vinceti, Salvatore Ferro, Jessica Mandrioli, ERRALS study group, Laura Ferri, Annalisa Gessani, Rocco Liguori, Pietro Cortelli, Roberto Michelucci, Fabrizio Salvi, Ilaria Bartolomei, Anna Maria Borghi, Andrea Zini, Rita Rinaldi, Valeria Tugnoli, Maura Pugliatti, Luca Codeluppi, Franco Valzania, Filippo Stragliati, Andi Nuredini, Sonia Romano, Alessandro D'Orsi, Liborio Parrino, Doriana Medici, Giovanna Pilurzi, Emilio Terlizzi, Donata Guidetti, Silvia De Pasqua, Mario Santangelo, Paola De Massis, Matteo Gizzi, Mario Casmiro, Pietro Querzani, Simonetta Morresi, Maria Vitiello, Marco Longoni, Alberto Patuelli, Susanna Malagù, Francesca Bianchi, Marco Currò Dossi, Cristiana Ganino

**Affiliations:** a Neuroscience PhD Program, University of Modena and Reggio Emilia, Modena, Italy; b Department of Neurosciences, Azienda Ospedaliero-Universitaria Di Modena, Modena, Italy; c Department of Pharmaceutical Sciences, Università degli Studi di Milano, via G. Colombo 71, 20133, Milan, Italy; d Associazione Farmaceutici dell'Industria (AFI), Viale Ranzoni 1, 20149, Milano, Italy; e Clinical and Experimental Medicine PhD Program, University of Modena and Reggio Emilia, Modena, Italy; f Department of Biomedical, Metabolic and Neural Sciences, University of Modena and Reggio Emilia, Modena, Italy; g Department of Neurology, St. Anna Hospital, Ferrara, Italy; h IRCCS Istituto delle Scienze Neurologiche di Bologna, Bologna, Italy; i Neurology Unit, Department of Neuroscience, University of Parma, Parma, Italy; j Unit of Neurosciences, Department of Medicine and Surgery, University of Parma, Parma, Italy; k Department of Neurology, IRCCS Arcispedale Santa Maria Nuova, Reggio Emilia, Italy; l Environmental, Genetic and Nutritional Epidemiology Research Center (CREAGEN), University of Modena and Reggio Emilia Medical School, Modena, Italy; m Department of Epidemiology, Boston University School of Public Health, Boston, USA; n Department of Hospital Services, Emilia Romagna Regional Health Authority, Bologna, Italy

**Keywords:** Amyotrophic lateral sclerosis, Tauroursodeoxycholic acid, Real-world evidence, Propensity score matching, Survival

## Abstract

**Background:**

Oral tauroursodeoxycholic acid (TUDCA) is a commercial drug currently tested in patients with amyotrophic lateral sclerosis (ALS) both singly and combined with sodium phenylbutyrate. This retrospective study aimed to investigate, in a real-world setting, whether TUDCA had an impact on the overall survival of patients with ALS who were treated with this drug compared to those patients who received standard care only.

**Methods:**

This propensity score–matched study was conducted in the Emilia Romagna Region (Italy), which has had an ALS regional registry since 2009. Out of 627 patients with ALS diagnosed from January 1st, 2015 to June 30th, 2021 and recorded in the registry with available information on death/tracheostomy, 86 patients took TUDCA and were matched in a 1:2 ratio with patients who received only usual care according to age at onset, sex, phenotype, diagnostic latency, ALS Functional Rating Scale-Revised (ALSFRS-R) at first visit, disease progression rate at first visit, and BMI at diagnosis. The primary outcome was survival difference (time from onset of symptoms to tracheostomy/death) between TUDCA exposed and unexposed patients.

**Findings:**

A total of 86 patients treated with TUDCA were matched to 172 patients who did not receive treatment. TUDCA-exposed patients were stratified based on dosage (less than or equal to 1000 mg/day or greater) and duration (less than or equal to 12 months or longer) of treatment. The median overall survival was 49.6 months (95% CI 41.7–93.5) among those treated with TUDCA and 36.2 months (95% CI 32.7–41.6) in the control group, with a reduced risk of death observed in patients exposed to a higher dosage (defined as ≥ 1000 mg/day) of TUDCA (HR 0.56; 95% CI 0.38–0.83; p = 0.0042) compared to both the control group and those with lower TUDCA dosages (defined as < 1000 mg/day). TUDCA was generally well-tolerated, except for a minority of patients (n = 7, 8.1%) who discontinued treatment due to side effects, primarily gastrointestinal and mild in severity; only 2 adverse events required hospital access but resolved without sequelae.

**Interpretation:**

In this population-based exploratory study, patients with ALS who were treated with TUDCA may have prolonged survival compared to patients receiving standard care only. Additional prospective randomized studies are needed to confirm the efficacy and safety of this drug.

**Funding:**

10.13039/501100009879Emilia-Romagna Region.

**Research in context**

**Evidence before this study**

To investigate tauroursodeoxycholic acid (TUDCA) effects in Amyotrophic Lateral Sclerosis (ALS) we conducted a non-systematic search in Pubmed including all studies published in English from Jan 1, 2017, to Jan 1, 2023, using the search terms “TUDCA” or “tauroursodeoxycholic” AND “amyotrophic lateral sclerosis” or “ALS”. Current evidence points to a protective effect of TUDCA in ALS, alone or combined with phenylbutyrate (PB) at dosages of 2000 mg/daily, in slowing disease progression and prolonging survival in post-hoc analysis of two phase II clinical trials. Phase III randomized clinical trials testing TUDCA and PB-TUDCA are currently ongoing, while in several countries PB-TUDCA received conditional approval for ALS treatment. TUDCA has been authorized as a medicinal product and is commercially available in food supplements; Therefore, it may be provided off-label upon evaluation by the rare disease technical group, in Emilia Romagna Region (Italy), where a regional registry prospectively collects clinical information of newly diagnosed ALS subjects since 2009.

**Added value of this study**

Observational studies provide real-world data (RWD) which could be helpful to fully understand the effects of the therapeutics in such rare diseases as ALS. With this propensity score-matched study, we analyzed TUDCA effects in terms of overall survival and safety in the general ALS population of Emilia Romagna Region, where ALS patients who were excluded from randomized clinical trials could be prescribed TUDCA since 2015 following approval from the regional rare disease technical group. Treatment with TUDCA at higher doses (more or equal to 1000 mg daily) resulted in prolonged survival compared to propensity score-matched non-TUDCA controls and patients assuming TUDCA at dosages inferior to 1000 mg daily. A minority of patients (n = 7, 8.1%) presented side effects, mainly involving the gastrointestinal tract and mild in severity, leading to treatment discontinuation; upon TUDCA withdrawal side effects vanished without leaving permanent sequelae.

**Implications of all the available evidence**

In the wider context of a regional ALS population, TUDCA is generally safe and well-tolerated and may have a beneficial effect in a fatal disease such as ALS by prolonging survival. These findings are not definitive and require additional supportive evidence by prospective randomized trials and larger worldwide post-marketing population studies.

## Introduction

Tauroursodeoxycholic acid (TUDCA) is an endogenously produced bile acid salt resulting from the conjugation of taurine to ursodeoxycholic acid. Apart from its primary use in the treatment of gallstones or primary cholestatic disease, there is a growing body of evidence, from in vitro and animal models, pointing to multiple mechanisms of neuroprotection in neurodegenerative diseases, including Amyotrophic Lateral Sclerosis (ALS). These mechanisms include the stabilization of mitochondrial membranes, enhancement of oxidative defenses, mitigation of endoplasmic reticulum stress, and modulation of the immune response, all of which may all contribute to TUDCA's cytoprotective and anti-apoptotic effects.^1^ A preliminary trial published in 2015 explored the safety and efficacy of TUDCA for ALS,^2^ where the primary outcome of the study (an improvement of at least 15% in the ALS Functional Rating Scale-Revised, ALSFRS-r, slope during the treatment period compared to the lead–in phase) was achieved.^2^ Since ALS is an incurable disease, the regional health care system of the Emilia Romagna Region (ERR) in Italy may support, following approval from the regional rare diseases technical group, the off-label use of approved treatments that show promising evidence from early clinical studies. In this context, since 2015, TUDCA has been prescribed by specialized ALS centers operating in ERR, that participate in ERR ALS register (ERRALS).^3^

We aimed to investigate, in a real-world setting through our ALS regional registry,^3^^,^^4^ whether tauroursodeoxycholic acid influences survival in the ALS population. Propensity score (PS) methods were adopted to correct for possible confounders that, in observational cohort studies, might impact on the outcome of interest,^5^ as in our case, survival.

## Methods

### Study design and setting

This is a retrospective, observational, propensity score-matched cohort study comparing patients with ALS treated with TUDCA with patients not exposed. The study was performed in ERR, where a prospective registry (ERRALS) has been active since 2009,^3^ collecting all incident ALS cases among residents, diagnosed according to Revised El Escorial Criteria (EEC-R).^6^

An electronic database is accessible only to the investigators of ERR Neurological Departments, where study referents upload a detailed phenotypic profile of new ALS cases. At diagnosis clinicians record baseline visits; follow-up is performed until death, collecting information regarding forced vital capacity (FVC), ALSFRS-R,^7^ drugs intake and discontinuation, riluzole and TUDCA assumption,^8^ supporting procedures,^9^ and cause, place and time of death.

In addition to demographic and clinical variables, for this study we extracted from ERRALS a detailed pharmacological history for TUDCA exposure, considering the maximum dosage, duration of treatment, and clinical variables at the time of TUDCA administration. Reasons for TUDCA discontinuation or dosage reduction were registered too. Data extraction for the last follow-up visit or death date was performed on 1st February 2022. The study was approved by the ethical committees of the coordinating centre and of ERR provinces (Comitato Etico Provinciale di Modena, number 124/08, 2 September 2008). Patients enrolled in ERRALS signed written informed consent. Neither clinicians nor patients received or were offered any compensation for the study. This study followed STROBE reporting guidelines.

### Study population

The study included all patients diagnosed with ALS from 1st January 2015 to 30th June 2021 recorded in ERRALS registry. The last date of follow-up was fixed on 1st February 2022. The start date for survival analysis was the date of disease onset. After the first visit, follow-up visits were recorded approximately every three months or based on patients’ needs, according to clinical practice for ALS.^10^

Patients were treated in the frame of regular ALS multidisciplinary clinical practice, and therapeutic decisions and medical care during the treatment were carried out by the treating physicians and were not defined by a specific protocol. Patients may or may not have received treatment with riluzole. TUDCA treatment was offered to patients with ALS not participating in other experimental trials at the time of the regular follow-up visits based on individual MND centre experience. Patients may or may not have continued TUDCA oral treatment until tracheotomy/death or censoring.

### Outcome measures

Safety analysis was performed on all TUDCA-exposed patients by recording reasons for discontinuation and possible side effects related to drug administration, hospitalizations, or excess mortality in TUDCA-treated patients. Cases receiving at least three consecutive months of daily TUDCA were included in propensity score matching (PSM) and effectiveness analysis; the matched control cohort was screened among non TUDCA-exposed patients registered in ERRALS during the same period as patients receiving TUDCA. The probability of receiving treatment with TUDCA was not influenced by economic or procedure-related variables, because the drug is covered by the ERR health system. Therefore, we estimated through stepwise logistic regression which clinical variables, also with a prognostic value, differed between treated and untreated patients: significant entering/excluding levels for variables were both set at 0.1. Based on these results, PSM was performed to reduce the bias due to confounding clinical variables by comparing outcomes among patients treated with or without TUDCA.^5^

The final model included sex, age at onset of ALS, diagnostic latency (disease duration from onset to diagnosis), ALS phenotype, body mass index (BMI), ALSFRS-R total score and disease progression rate calculated at first visit. Once the propensity scores were calculated, they were used for matching case and control groups. A greedy nearest-neighbor matching was adopted. A caliper of 0.2 standard deviations of the logit of the propensity score was set.^5^^,^^11^ The matching ratio between controls and cases was set at 2:1. Considering the limited number of TUDCA-treated patients in the database, such a ratio was selected to increase in statistical power given the expected prevalence of exposure among the controls.

The primary outcome to evaluate TUDCA effectiveness was survival probability considering tracheostomy-free survival at the end of the study. The secondary outcomes included disease progression during the follow-up period, which was measured by the monthly decline in ALSFRS-R at the last visit. This decline was calculated by dividing the difference in the ALSFRS-R total score between the initial visit and the last available follow-up before tracheostomy or death (whichever occurred first) by the number of months between the two visits. Finally, we also assessed the frequency and time to NIV and PEG in patients treated with TUDCA compared with untreated patients.

Adherence to treatment with TUDCA was assessed by calculating the rate of treatment discontinuation and the reason.

### Statistical analysis

For analyses, the disease progression rate at diagnosis was assessed by subtracting the ALSFRS-R total score at the time of diagnosis from the presumed score of 48 at onset. The obtained difference was then divided by the duration of diagnostic delay in months. Since 48 represents the highest achievable score on the ALSFRS-R scale, we assume that each patient had this score before the onset of symptoms.^12^

Given that in the registry, any resident patient diagnosed and followed up within the specialized Motor Neuron Disease (MND) centres of Emilia Romagna was documented with ALSFRS-r at the time of diagnosis, the disease progression rate at diagnosis aligns with the disease progression rate at the first visit. The ALS progression rate at the last visit was calculated by dividing the difference in ALSFRS-r scores between the last and first visits by the time interval in months between the two visits.

Survival was calculated by considering the time in months from symptom onset to death or tracheostomy (whichever occurred first) or the censoring date (the last day of follow-up, February 1st, 2022).

Survival and progression rates were taken into account to classify the patient's disease progression as “slow,” “intermediate,” or “fast.” We classified patients as slow progressors if individuals had tracheostomy-free survival exceeding 60 months from symptom onset, or if living patients (at their latest observation) demonstrated tracheostomy-free survival of >24 months and a disease progression rate of <0.3 points per month, measured from onset to the initial visit. We designated fast progressors as individuals with tracheostomy-free survival of less than 24 months from symptom onset or living patients (at their most recent observation) displaying tracheostomy-free survival <60 months and a disease progression rate >1.3 points per month, measured from onset to the initial visit. All remaining patients were categorized as intermediate progressors.

Indicator variables were created to provide a stratification of TUDCA treatment, in terms of strengths and duration. TUDCA strength was defined as a three-level categorical variable: ‘‘no drug’’, ‘‘TUDCA < 1000 mg/day”, “TUDCA ≥ 1000 mg/day”. Duration of TUDCA treatment was classified according to time intervals of 12 months (“no drug”, “TUDCA < 12 months”, “TUDCA ≥ 12 months”), based on the distribution of TUDCA-treated patients (40 patients assumed TUDCA for less than 12 months, 44 for more or equal than 12 months). For 2 TUDCA-treated patients, the duration of the therapy could not be established and were excluded from the analysis.

After PSM, the balance between the case/control group was evaluated by calculating standardized mean differences (SMDs) between treated and matched control patients in the matched and overall populations. SMDs of less than 10% were considered negligible imbalances.

Description of treatment groups homogeneity was presented using Student's t-test or ANOVA and chi-square test, when appropriate. Survival was calculated from onset to death/tracheostomy or the censoring date using the Kaplan–Meier method; the logrank test was used for group comparison. The hierarchy of performed statistical analyses was the following: i) impact of TUDCA treatment on survival; ii) impact of treatment duration on survival; iii) impact of TUDCA doses on survival. In parallel, multivariate Cox regression analysis was employed to obtain a better estimation of survival probabilities of TUDCA-treated patients versus control. Since the delay in the initiation of TUDCA administration may constitute an immortal time bias,^13^ we included this variable as a covariate in Cox regression analysis; whereas Riluzole and respiratory function were included although not unbalanced between TUDCA groups. These analyses were repeated in a sub-cohort of patients in which TUDCA <1000 mg/day and their matched controls were excluded.

Missing data were not substituted, and they were treated as such. For primary outcomes, no missing data were reported in the database; lost in follow-up were censored recording patients as alive at the last available visit. For the ALS progression rate at last visit, 26 values were missing, exclusively in the non-TUDCA group; then, the pairwise analyses were performed excluding paired data with missing values.

Calculations were performed with SAS 9.4 (2021).

### Role of the funding source

The funder of the study had no role in study design, data collection, data analysis, data interpretation, or writing of the report. All authors had access to the data and were responsible for the decision to submit for publication.

## Results

### Clinical characteristics of patients with ALS before propensity score matching

The initial population considered for the study was constituted of 627 patients (men: women = 1.36), whose clinical features are shown in Supplementary Table S1. Median survival time was 25.5 months (95% CI 28.7–31.8) from onset.

Patients exposed to TUDCA (n = 86) were more commonly men and on average they were less likely to present bulbar onset/phenotype, while they frequently had a younger-onset disease and more benign phenotypes such as flail arm/leg and UMN-predominant. Both MiToS (median 0, IQR 0–1) and King's College (median 2, IQR 1–3) stages were low at TUDCA initiation, suggesting exposed patients were at the initial stages of the disease.

### Propensity score matching

TUDCA-treated and non-TUDCA-treated patients with ALS were matched for sex, age at onset, ALSFRS-R, diagnostic delay, phenotype, BMI, and disease progression rate at diagnosis. After matching, SMD tests confirmed the balance between the case/control group calculated by PSM for almost all clinical characteristics (Supplementary Table S2). As reported in Table 1, no statistically relevant differences were observable for clinical characteristics in the TUDCA-treated and non-TUDCA-treated patients, suggesting a good homogeneity of the cohort.

**Table 1:** Demographic and clinical characteristics TUDCA-treated and non-TUDCA-treated patients with ALS after matching.

Clinical features	Patients not treated with TUDCA (n = 172) n (%) m [SD]	Patients treated with TUDCA (n = 86) n (%) m [SD]	p-value
Sex, n (%)			0.77
Male	125 (72.7)	64 (74.4)	
Female	47 (27.3)	22 (25.6)	
Months from onset to diagnosis, mean [SD]	11.6 [8.2]	11.9 [10.2]	0.78
Age at onset, mean [SD]	58.1 [10.8]	58.2 [9.3]	0.97
Site of onset, n (%)			0.95
Bulbar	38 (22.1)	19 (22.1)	
Upper limbs	72 (41.9)	35 (40.7)	
Lower limbs	59 (34.3)	31 (36.0)	
Respiratory	3 (1.7)	1 (1.2)	
*Phenotype*			0.94
Bulbar	38 (22.1)	18 (20.9)	
Classic	99 (57.6)	52 (60.5)	
Flail arm and flail leg	28 (16.3)	12 (14.0)	
UMN-p	4 (2.3)	3 (3.49)	
Respiratory	3 (1.7)	1 (1.16)	
Familial ALS, n (%)	15 (8.7)	10 (11.6)	0.46
BMI at diagnosis, mean [SD]	24.4 [4.0]	24.7 [3.7]	0.59
ALSFRS-r at diagnosis, mean [SD]	41.7 [4.8]	41.8 [5.1]	0.86
Disease progression rate at diagnosis, mean [SD]	0.648 [0.589]	0.626 [0.650]	0.79
FVC at diagnosis, mean [SD]	93.5 [22.0]	92.9 [23.9]	0.89
FTD at diagnosis, n (%)	11 (6.4)	6 (7.0)	0.86
Riluzole, n (%)	152 (88.4)	82 (95.4)	0.069
MiToS stage at TUDCA beginning [SD]	–	0.58 [0.89]	–
King's stage at TUDCA beginning [SD]	–	2.26 [0.96]	–
Months from onset to TUDCA intake, mean [SD]	–	22.8 [16.4]	–
Months from diagnosis to TUDCA intake, mean [SD]	–	11.0 [10.7]	–
Absolute duration of TUDCA treatment in days, mean [SD]	–	449 [392]	–

SD: Standard Deviation; UMN-p: Upper Motor Neuron predominant; BMI: Body Mass Index; ALSFRS-R: ALS Functional Rating Scale—Revised; FVC: Forced Vital Capacity; FTD: Frontotemporal Dementia. Based on the nature of the clinical variable under investigation, homogeneity between cases and controls was assessed by the Student's T test or chi-square test.

The resulting p-values are representative of good homogeneity of clinical features among tested groups.

### TUDCA treatment

**Fig. 1:**
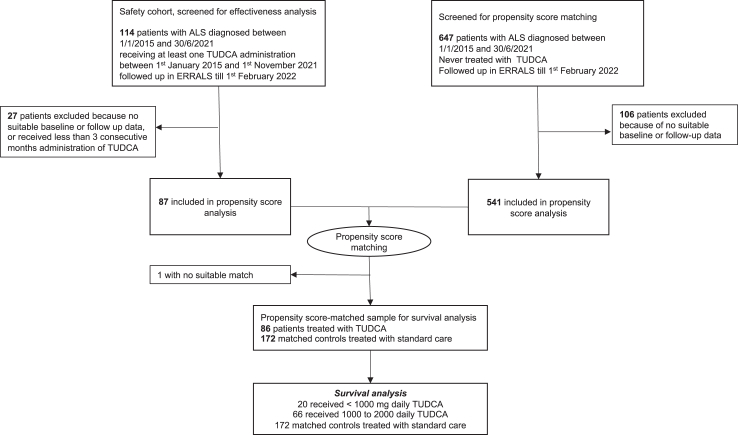
**Participant flow through study analysis**. Cases and controls of the ERRALS study population are shown through the flowchart. 761 patients were diagnosed with ALS between January, 1st, 2015 and June, 30th, 2021 in the Emilia Romagna region and recorded in ERRALS. Last follow-up date was fixed on February 1st, 2022. All patients with ALS with insufficient baseline or follow-up data on the ERRALS registry were excluded from propensity score-matching analysis; TUDCA-exposed patients who took less than three consecutive months of therapy were similarly excluded. ALS: Amyotrophic Lateral Sclerosis; ERRALS: Emilia Romagna Region ALS registry; TUDCA: tauroursodeoxycholic acid.

After applying the PSM method, 86 TUDCA-treated cases were matched to 172 non-TUDCA-treated ALS and were included in the analysis (Fig. 1). Initiation of TUDCA treatment occurred after a median of 17.7 months (IQR: 10–31.5) since onset and 7.8 months (IQR: 2.4–17) since diagnosis. Median TUDCA treatment duration was 12.6 months (IQR: 7.2–17). All patients who stopped TUDCA treatment before 6 months (n = 13, 15.1%) remained in the database.

TUDCA was administered at different dosages to patients. Table 2 shows the clinical features of patients stratified based on TUDCA dosages.

**Table 2:** Clinical characteristics of patients with ALS stratified according to exposure to different dosages of TUDCA.

Clinical features	Patients treated with TUDCA <1000 mg/day (n = 20) n (%), m [SD]	Patients treated with TUDCA ≥1000 mg/day (n = 66) n (%), m [SD]	p-value
Sex, n (%)			0.60
Male	14 (70.0)	50 (75.8)	
Female	6 (30.0)	16 (24.2)	
Months from onset to diagnosis, mean [SD]	11.5 [7.9]	12.1 [10.9]	0.82
Age at onset, mean [SD]	61.7 [10]	57.1 [8.9]	0.055
Site of onset, n (%)			0.054
Bulbar	7 (35)	12 (18.2)	
Upper limbs	3 (15)	32 (48.5)	
Lower limbs	10 (50)	21 (31.3)	
Respiratory	–	1 (1.5)	
Phenotype			0.28
Bulbar	7 (35)	11 (16.7)	
Classic	10 (50)	42 (63.6)	
Flail arm and flail leg	3 (15)	9 (13.6)	
UMN-p	–	3 (4.6)	
Respiratory	–	1 (1.5)	
BMI at diagnosis, mean [SD]	25.3 [4.8]	24.5 [3.4]	0.54
ALSFRS-r at diagnosis, mean [SD]	40.3 [5.5]	42.3 [4.9]	0.12
Disease progression rate at diagnosis, mean [SD]	0.82 [0.7]	0.57 [0.6]	0.12
FVC at diagnosis, mean [SD]	87 [22.6]	95.1 [24.3]	0.35
FTD presence at diagnosis, n (%)	2 (10)	4 (6.1)	0.54
Riluzole, n (%)	20 (100)	62 (93.9)	0.26
MiToS stage at TUDCA beginning [SD]	0.80 [1]	0.51 [0.84]	0.21
King's stage at TUDCA beginning [SD]	2.50 [1.05]	2.18 [0.93]	0.20
Months from onset to TUDCA intake, mean [SD]	19.8 [14.4]	23.7 [17]	0.36
Absolute duration of TUDCA treatment in days, mean [SD]	365 [376]	474 [396]	0.28
Drug titration, n (%)	9 (45)	47 (71.2)	**0**.**034**
Drug discontinuation, n (%)	5 (25)	11 (16.7)	0.40

SD: Standard Deviation; UMN-p: Upper Motor Neuron predominant; BMI: Body Mass Index; ALSFRS-R: ALS Functional Rating Scale—Revised; FVC: Forced Vital Capacity; FTD: Frontotemporal Dementia.

Based on the nature of the clinical variable under investigation, the homogeneity between cases and controls was assessed by ANOVA or chi-square test.

The resulting p-values are representative of good homogeneity of clinical features among tested groups.

p-values inferior to 0.05 are reported in bold character.

### Survival in TUDCA-treated patients compared with non-TUDCA-treated patients

**Fig. 2:**
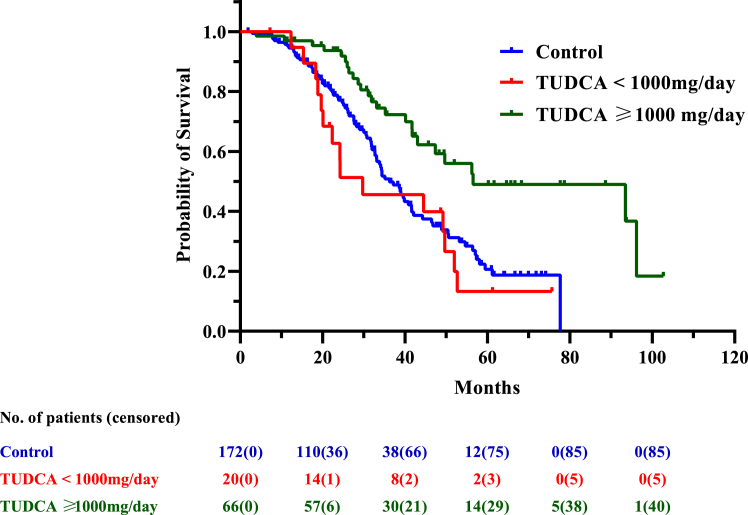
**Tracheostomy-free/life survival from diagnosis of incident ALS cases based on TUDCA treatment**. Kaplan–Meier survival plot displaying tracheostomy-free survival of patients with ALS in ERRALS study divided by controls (i.e., non-TUDCA-exposed patients, blue line), low-dose TUDCA-exposed patients (i.e., <1000 mg/daily dosage, red line), and high-dose TUDCA patients (≥1000 mg/daily dosage, green line). Survival was calculated by considering the time in months from symptom onset to death or tracheostomy (whichever occurred first) or the censoring date (the last day of follow-up, February 1st, 2022). The number of patients included in the analysis is presented every 20 months, with the number of censored patients between brackets. TUDCA: tauroursodeoxycholic acid.

During the follow-up (median 41.56 months [IQR, 26.33–77.65 months]) 40 deaths/tracheostomy (46.5%) were recorded among the TUDCA-treated cases compared with 87 events (50.6%) among the non-TUDCA-treated controls. Higher dosage TUDCA-treated patients had longer survival (median: 56.5 months, 95% CI 43.0–not available) compared to both lower doses-TUDCA (median survival: 29.7 months, 95% CI 19.6–49.6 months) and non-TUDCA treated patients (median: 36.2 months, 95% CI 32.7–41.6 months) (Table 3, Fig. 2).

**Table 3:** Median survival and hazard ratio on different dosages and durations of TUDCA treatment.

Group	Survival (months)		Hazard ratio		
	Median (CI)	Log-Rank	HR	95% CI	p-value
**Survival Analyses (from onset to death/tracheotomy) performed on PSM cohort**					
***Level I: Survival: from onset to death/tracheotomy (treatment impact)***					
Patients not treated with TUDCA (n = 172)	36.2 (32.7–41.6)	–	–	–	–
Patients treated with TUDCA (n = 86)	49.6 (41.7–93.5)	**0**.**0062**	0.57	0.38–0.83	**0**.**0042**
***Level II: Survival: from onset to death/tracheotomy (duration impact)***					
Patients not treated with TUDCA (n = 172)	36.2 (32.7–41.6)	–	–	–	–
Patients treated with TUDCA:					
• With TUDCA <12 months (n = 40)	44.5 (26.3-NA^a^)	0.23	0.73	0.44–1.22	0.23
• With TUDCA >12 months (n = 44)	56.2 (43–96.2)	**0**.**0022**	0.67	0.52–0.87	**0**.**0024**
***Level III: Survival: from onset to death/tracheotomy (dosage impact)***					
Patients not treated with TUDCA (n = 172)	36.2 (32.7–41.6)	–	–	–	–
Patients treated:					
• With TUDCA <1000 mg/day (n = 20)	29.7 (19.6–49.6)	0.16	1.10	0.64–1.92	0.72
• With TUDCA ≥1000 mg/day (n = 66)	56.5 (43.0-NA^a^)	**<0**.**0001**	0.42	0.26–0.68	**<0**.**0001**
**Analyses performed on sub PSM cohort (excluding TUDCA < 1000 mg/day and their matched controls)**					
**Survival: from onset to death/tracheotomy (treatment impact)**					
Patients not treated with TUDCA (n = 132)	39.0 (33.1–48.8)				
Patients treated with ≥1000 mg/day (n = 66)	56.5 (43.0-NA^a^)	**0**.**0019**	0.45	0.28–0.73	**0**.**0011**

NA: not available. CI: confidence interval. HR: Hazard Ratio. FVC: forced vital capacity.

Median survival times from Kaplan–Meier analyses and Hazard Ratio descriptors from multiple Cox regression analyses were reported, respectively. For the latter ones, the following covariates were included in the model: riluzole treatment; delay of TUDCA initiation from onset; delay of TUDCA initiation from diagnosis; and FVC value at the baseline. Supplementary Table S3 reported p-values to estimate the impact of such covariates in terms of confounding factors.

p-values inferior to 0.05 are reported in bold character.

a The CI cannot be estimated because a low observations' size or a higher number of censored observations.

Since patients treated with lower doses of TUDCA were on average older, more advanced in disease and more often bulbar (Table 2), to avoid a confounding effect of these factors, we performed survival analysis after the removal of lower doses TUDCA-exposed patients (n = 20) and their matched unexposed counterpart (n = 40), leaving 66 patients exposed to higher than 1000 mg daily TUDCA with 132 matched controls. Even comparing these two populations, TUDCA was found associated with a reduced risk of death/tracheostomy by 55% (HR 0.45, 95% CI 0.28–0.73, p = 0.0011). Stratification by duration revealed patients exposed to TUDCA for more than 12 months had prolonged survival (median: 56.2 months, 95% CI 43.0–96.2) compared to non-TUDCA patients (median: 36.2 months, 95% CI 32.7–41.6). High-dose TUDCA treatment yielded superior results in terms of survival both versus controls (Table 3) and low-dose treatment (HR 0.38, 95% CI 0.2–0.74, p < 0.0001).

Multiple Cox regression analysis confirmed the prognostic impact of this covariate (HR = 0.94, 95% CI 0.92–0.97, p < 0.0001), while TUDCA delay since diagnosis was non-relevant (HR: 1.01, 95% CI 0.98–1.04, p = 0.59). Inclusion of the possible confounding variables in covariate analysis revealed riluzole did not act as a potential confounder in Cox regression analysis, whereas FVC at baseline did (Supplementary Table S3). In the same analysis delay of TUDCA initiation since diagnosis did not affect survival, contrarily to the delay of treatment initiation since onset (Supplementary Table S3).

Median time from onset to NIV initiation was 30.9 months in TUDCA-treated patients (95% CI: 19.1–41.0) and 26.2 months (95% CI 21.9–29.9) in non-treated patients (HR 0.73; 95% CI 0.43–1.24, p = 0.2).

Considering only high dose TUDCA-treated patients (n = 66) and their matched controls (n = 132) median time from onset to NIV initiation was 36.7 months in TUDCA-treated patients (95% CI: 31.1–56.1) and 29.9 months (95% CI 25.0–34.1) in non-treated patients (HR 0.65; 95% CI 0.44–0.70, p = 0.034).

Onset to PEG positioning occurred after a median of 23.2 months in TUDCA-treated patients (95% CI 20.3–27) and 24.8 months (95% CI 21.3–31.7) in non-treated patients (HR 1.25; 95% CI from 0.551 to 2.85, p = 0.65). Disease progression rate at last visit was −1.11 points/month (95% CI, −0.93 to −1.29 points/month) for patients non-treated with TUDCA (n = 146) compared with −0.98 points/month (95% CI, −0.69 to −1.27 points/month) for TUDCA-treated patients (n = 86) (p = 0.44).

### Survival in high dose TUDCA-treated patients compared with matched non-TUDCA-treated patients on specific ALS subgroups

Survival analyses were performed separately on specific patients’ subgroups comparing high-dose (≥1000 mg/day) TUDCA-treated patients (n = 66) with propensity score-matched patients who were not treated with TUDCA (n = 132).

Among slow progressors (n = 75), the median time from onset to death or tracheostomy was 96.16 months (95% CI: 93.47 to not available) in high TUDCA-treated patients and 57.34 months (95% CI: 41.56 to not available) in non-treated patients (HR 0.17; 95% CI 0.05–0.60, p = 0.0065).

Among fast progressors (n = 41) and intermediate progressors (n = 82) there was no difference in survival between high-dose TUDCA-treated patients and propensity score matched non-treated patients (Supplementary Table S4). When considering fast and intermediate progressors altogether, a difference was detected (Supplementary Table S4).

There were no differences in survival among bulbar patients (n = 41) based on TUDCA treatment: median time from onset to death or tracheostomy was 31.82 months (95% CI: 24.49 to not available) in high-dose TUDCA-treated patients and 34.09 months (95% CI: 22.42–44.22) in propensity-matched non-treated patients (HR 0.81; 95% CI 0.26–2.49, p = 0.71).

Among spinal onset patients (n = 157), median time from onset to death or tracheostomy was 93.47 months (95% CI: 43.04–96.16) in high-dose TUDCA-treated patients and 41.55 months (95% CI: 33.07–50.53) in propensity matched non-treated patients (HR 0.44; 95% CI 0.26–0.76, p = 0.0029) (Supplementary Table S4).

When survival analysis was performed separately on familial ALS, we could not detect any significant effect of TUDCA administration, though these results are limited by the small number of familial patients with ALS in our cohort (only 20 individuals in the second propensity-matched cohort). Similar results were obtained with survival analyses on *C9ORF72* expanded patients (Supplementary Table S4).

### Adverse effects in TUDCA-treated patients

Eighteen patients (20.9%) presented side effects (n = 20) requiring drug reduction (n = 13, 15.1% of total TUDCA exposed patients) or drug discontinuation (n = 7, 8.1% of total TUDCA patients); two (2/13, 15.4%) of these patients initially reduced the dose to cope with side effects but eventually decided to drop treatment. The most frequently reported adverse effects were diarrhoea (n = 12; 14.0%), abdominal pain (n = 5; 5.8%), and skin eruption (n = 3; 3.5%). Among the seven patients who discontinued TUDCA (38.9% of patients presenting side effects), three presented diarrhoea, two abdominal pain and two skin eruptions; two of these events required hospital access but resolved without sequelae after treatment discontinuation. No deaths or abnormal increase in disease progression was observed for TUDCA-exposed patients. No differences were noted concerning the onset of adverse effects among patients receiving varying dosages of TUDCA (number of side effects: 5 out of 20 patients treated with TUDCA <1000 mg/day, and 15 out of 66 patients treated with the higher dose). In general, patients exposed to higher doses of TUDCA were more frequently subject to drug titration compared to low-dose exposed TUDCA patients (71% versus 45%, p = 0.03). Overall, 16 patients discontinued TUDCA treatment (18.6% of total TUDCA patients); besides adverse events, three patients (3.50%) decided to interrupt TUDCA administration because of lack of observable effects, four (4.65%) because of engagement in other clinical trials, and finally one (1.16%) because of progressive swallowing problems which rendered TUDCA administration difficult.

## Discussion

This multicenter, propensity score-matched cohort study suggested through real-world data that patients with ALS treated with TUDCA may have a survival benefit. As far as the TUDCA effect is concerned in randomized clinical trials (RCT), after the first exploratory Italian study showing encouraging results in ALS^2^ a phase III study is still going on in Europe.^14^ Moreover, a combination of TUDCA and sodium phenylbutyrate (PB) gave promising effects both on the ALSFRS-R decline^15^ and on survival^16^^,^^17^ leading to conditional approval of the drug outside Europe, where instead a phase III trial is still ongoing.

While evidence from phase III RCTs of the effects of TUDCA alone (ClinicalTrials.gov Identifier: NCT03800524) or in combination with phenylbutyrate (PB) (ClinicalTrials.gov Identifier: NCT05021536) is awaited, a potential advantage given by population-based studies is the possibility to examine treatment effects for a longer time (as in our case, superior to one year) and consider patients at all stages of disease while assuming approved drugs for the disease. This will avoid the selection of a restricted study population that might not be entirely representative of the overall ALS population.^18^ In special fragile populations such as patients with ALS several comorbidities may coexist with an impact on survival,^19^^,^^20^ leading to patients’ exclusion from RCT because of stringent inclusion and exclusion criteria^21^ and consequently a gap in the generalizability of the results.^22^ To favour the inclusion of population-based data during the regulatory approval process, a framework for evaluating the use of real-world evidence (RWE) was created in the US.^23^ Real-world studies are now being considered to generate RWE of both safety and effectiveness, and to support regulatory decisions about drug products; in ALS RWE generated interesting data on controversial drugs such as edaravone as well.^24^^,^^25^

In our retrospective propensity score matched study, we found that treatment with TUDCA was associated with reduced risk of death and/or tracheotomy over 50% in patients treated with daily doses ≥1000 mg in comparison to controls. Importantly, this effect was observed in a limited, population-based cohort, where spinal/bulbar/respiratory onset patients with ALS were included in the analysis, differently from RCTs where rarer phenotypes might be excluded because of stringent exclusion criteria. Treatment with lower doses of TUDCA did not affect survival in comparison to controls, in agreement with the proposed therapeutical dosage tested in previous studies,^2^^,^^15^ establishing a threshold effect.

Though in our PSM population-based cohort riluzole seemed not to act as a potential confounder in survival analysis, it should be noted a higher percentage of TUDCA-exposed patients concurrently assumed riluzole, similar to ongoing RCTs where the combination of the two is studied.

Should our results be confirmed in further prospective studies this will open a new scenario, where establishing the effect of TUDCA alone or in combination with PB would be of uttermost importance. Despite our observed trend towards a slowing of disease progression in patients treated with TUDCA, we could not demonstrate a significant effect of the drug on ALSFRS-R monthly decline. This can be partly explained by the retrospective nature of this study and several potential limits of clinical data reliability in population registries: the irregular intervals between each visit and consequently ALSFRS-r collection time points, ALSFRS-r inter-rater differences among population MND clinics, and the loss in follow-up. However, recent studies proved a high variability in disease trajectories exists in ALS,^26^ destabilizing the significance given so far to ALSFRS-r slope in clinical trials. In fact, newer clinical outcomes derived from statistical enrichment techniques have been tested and are the subject of research for a better definition of efficacy endpoints in clinical trials as well as population studies.^27^^,^^28^

When comparing only high-dose TUDCA-treated patients with propensity score-matched untreated patients, we also identified a significant difference in the time from onset to initiation of non-invasive ventilation (NIV), which may suggest a potential beneficial effect of TUDCA in slowing down disease progression, particularly in its early stages. Our survival analysis within subgroups revealed a noteworthy treatment effect on individuals with slow progression; however, this effect was not evident among those with intermediate and fast progression, unless these groups were analyzed collectively. Spinal onset patients also benefited most from TUDCA treatment compared to bulbar onset patients with ALS. Given the limited number of patients within each subgroup, the generalizability of these findings is constrained, warranting caution when interpreting survival data. These observations, together with results on other patients’ subgroups based on site of onset, family history, or genetics, necessitate confirmation through prospective randomized controlled trials, where instrumental evaluations such as FVC are regularly assessed and close monitoring of fast progressors due to the rapid evolution of their condition is granted.

Our study showed that the safety profile of TUDCA, even with a longer treatment duration, was mainly characterized by gastrointestinal side effects reported in almost 20% of TUDCA-exposed patients, independently of BMI. Drug reduction was sufficient for most patients presenting with side effects, but 35% of them (corresponding to 7% of the entire treated cohort) judged these effects intolerable and discontinued the treatment. Therefore, TUDCA treatment has shown to be well tolerated in real-world settings where patients with ALS may experience a large range of comorbidities potentially increasing side effects and affecting survival, and several other treatments potentially modulating drug interactions.^19^ Patients assuming higher doses of TUDCA were more frequently exposed to increasing titration regimens, which may be a good therapeutic strategy to cope with unwanted effects and to establish the maximum tolerated TUDCA dose in individual patients.

Due to the observational nature of our study, the accurate drug history and adherence to TUDCA treatment could only be verified by the attending physician during follow-up visits. The absence of external drug accountability controls, as expected in randomized controlled trials, could potentially influence the results. Moreover, vitamins, supplements, and other possible medications consumed by patients, which were not monitored, could introduce confounding in an observational study like ours. The same consideration applies to socioeconomic factors, dietary habits, or other health-related behaviours, as these data were not accessible and may have contributed to selection bias in individuals who opt to take substances like TUDCA, which could also coincide with other practices exerting a favourable influence on the disease.

As part of the limitations of our study, the already-mentioned retrospective observational design, which entails irregular follow-ups, missing and non-uniform information for example for clinical scales (ALSFRS-r) or instrumental measures (FVC), impossibility to fully remove possible confounding factors, and, most importantly, immortal time bias when survival analysis is among prespecified outcome analysis.^29^ Nevertheless, our regional registry gathers a large amount of clinical information about patients with ALS, regularly verified and controlled by trained clinicians, allowing for a more accurate propensity score analysis of patients with ALS compared to previous reports.^24^^,^^25^

Finally, the exploratory nature of the study, coupled with the limited sample size and underpowered analyses, prevents us from making conclusive determinations regarding outcome measures, especially concerning the assessment of adverse effects.

In summary, in our “real-world” study patients who received TUDCA at the higher dose survived longer. Since this was not a controlled study, we cannot rule out additional confounding factors but pending the results of ongoing phase III RCTs, this study seems to confirm current evidence from early trials. Our propensity score matched cohort study offers the advantage of longer follow-up than RCTs, allowing to measure survival, the most clinically meaningful outcome measure for assessing the benefits of ALS treatments,^30^ although almost disused as the primary outcome in ALS trials.^22^ Further ongoing studies (NCT03800524, NCT05021536) will be crucial to verify TUDCA effects alone or in combination with PB.

## Contributors

EZ and JM had full access to all the data in the study and take responsibility for the integrity of the data and the accuracy of the data analysis. EZ and UM contributed equally as co−first authors. JM and GF contributed equally as co–senior authors.

*Concept and design*: EZ, JM, GF, UM, IM.

*Acquisition, analysis, or interpretation of data*: All authors.

Data verification:UM; JM.

*Drafting of the manuscript*: EZ, JM, GF, UM.

*Critical revision of the manuscript for important intellectual content*: MV, IM, NF, CS, GG, VV.

*Statistical analysis*: EZ, UM, JM.

*Administrative, technical, or material support: SF, J*M.

*Supervision*: EZ, JM, GF.

## Data sharing statement

With publication, deidentified data and data dictionary will be shared with researchers who provide a methodologically sound proposal and will include investigator support to achieve aims in the approved proposal. Proposals should be directed to elisabetta.zucchi@unimore.it or jessica.mandrioli@unimore.it; to gain access, data requestors will need to sign a data access agreement. Proposals may be submitted up to 36 months following article publication. After 36 months, the data will be available in our university's data warehouse but without investigator support other than deposited metadata.

## Declaration of interests

EZ, UM, GF, IM, CS, GG, NF, AG, MC, ES, LZ, PA, EC, MV, SF, VV declare no conflicts of interest. JM reports receiving advisory board fees from Biogen, Amylix and Italfarmaco, grant support from Roche and from Pfizer (RAP-ALS study; drug furniture), all unrelated to this study. JM received grant support from Agenzia Italiana del Farmaco [grant number 2016–02364678], Italian Ministry of Health (bando per la ricerca finalizzata 2016, grant number RF-2016-02361616), and University of Modena and Reggio Emilia (bando FAR 2021, Progetti di ricerca Interdisciplinari Mission Oriented, NEURALS project), all unrelated to this study.

## Appendix A Supplementary data

Supplementary material

ERRALS group for PUBMED

Italian abstract
