# Supplementary material for: Effect of tauroursodeoxycholic acid on survival and safety in amyotrophic lateral sclerosis: a retrospective population-based cohort study
Source: eClinicalMedicine. 2023 Oct 5;65:102256. doi: 10.1016/j.eclinm.2023.102256 (PMC10570688; doi:10.1016/j.eclinm.2023.102256)
Supplement: Supplementary material [file mmc1.pdf]

SUPPLEMENTARY MATERIAL

Effect of tauroursodeoxycholic acid on survival and safety in amyotrophic lateral sclerosis: a retrospective population-based cohort study

Elisabetta Zucchi[1,2*], MD; Umberto Maria Musazzi[3*], PhD; Guido Fedele[4*], MSc; Ilaria Martinelli[1,5], MD; Giulia Gianferrari[1,6], MD; Cecilia Simonini[1], BSc; Nicola Fini[1], MD; Andrea Ghezzi[1,6], MD; Maria Caputo[1,6], MD; Elisabetta Sette[7], MD; Veria Vacchiano[8], MD; Lucia Zinno[9], MD; Pietro Anceschi[10], MD; Elena Canali[11], MD; Marco Vinceti[12,13], MD; Salvatore Ferro[14], MD; Jessica Mandrioli[1,6], MD, Prof; for ERRALS study group.

(1) Department of Neurosciences, Azienda Ospedaliero-Universitaria Di Modena, Modena, Italy.
(2) Neuroscience PhD Program, University of Modena and Reggio Emilia, Modena, Italy.
(3) Department of Pharmaceutical Sciences, Università degli Studi di Milano, via G. Colombo 71, 20133 Milan, Italy.
(4) Associazione Farmaceutici dell'Industria (AFI), Viale Ranzoni 1, 20149 Milano, Italy.
(5) Clinical and Experimental Medicine PhD Program, University of Modena and Reggio Emilia, Modena, Italy.
(6) Department of Biomedical, Metabolic and Neural Sciences, University of Modena and Reggio Emilia, Modena, Italy.
(7) Department of Neurology, St. Anna Hospital, Ferrara, Italy
(8) Department of Biomedical and Neuromotor Sciences, University of Bologna and IRCCS Istituto delle Scienze Neurologiche di Bologna, Bologna, Italy
(9) Department of Neuroscience, Neurology Unit, University of Parma, Parma, Italy
(10) Department of Medicine and Surgery, Unit of Neurosciences, University of Parma, Parma, Italy
(11) Department of Neurology, IRCCS Arcispedale Santa Maria Nuova, Reggio Emilia, Italy
(12) Environmental, Genetic and Nutritional Epidemiology Research Center (CREAGEN), University of Modena and Reggio Emilia Medical School, Modena, Italy
(13) Department of Epidemiology, Boston University School of Public Health, Boston - USA
(14) Department of Hospital Services, Emilia Romagna Regional Health Authority, Bologna, Italy

**these authors contributed equally to the study.*

Corresponding author:
Jessica Mandrioli, MD
Department of Biomedical, Metabolic and Neural Sciences, University of Modena and Reggio Emilia,
Via Pietro Giardini n. 1355
41100 Modena, Italy
Tel. 00390593961640 Fax 00390593963775
Cell +303392633246
E-Mail jessica.mandrioli@aou.mo.it

INDEX

Supplementary Table 1……………………………………………………………………3

Supplementary Table 2……………………………………………………………………4

Supplementary Table 3……………………………………………………………………5

Supplementary Table 4……………………………………………………………………6

Supplementary Appendix…………………………………………………………………7

Supplementary Table 1: Demographic and clinical characteristics of patient population (before matching) (N = 627)

Characteristics	**Oral TUDCA treatment**		**p-value**
	**Yes<br>N=86<br>n (%)<br>m [SD]**	**No<br>N=541<br>n (%)<br>m [SD]**	
**Sex, male**	64 (74·4)	297 (54·9)	**0·0006**
**Age at onset, years**	58·2 [9·3]	67·0 [11·4]	**< 0·0001**
**Diagnostic delay, months**	11·9 [10·2]	13·4 [11·3]	0·25
**Site of onset**			0·15
Bulbar	19 (22·1)	187 (34·6)	
Upper limb	35 (40·7)	166 (30·7)	
Lower limb	31 (36·0)	178 (32·9)	
Respiratory	1 (1·2)	10 (1·8)	
**Phenotype**			0·45
Bulbar	18 (20·9)	180 (33·3)	
Classic	52 (60·5)	269 (49·7)	
Flail arm and leg	12 (14·0)	48 (8·9)	
UMNp	3 (3·5)	35 (6·5)	
Respiratory	1 (1·2)	9 (1·7)	
**Revised El Escorial criteria**			0·12
Definite	22 (25·6)	181 (33·4)	
Clinically probable	28 (32·6)	173 (32·0)	
Probable lab-supported	16 (18·6)	74 (13·7)	
Possible	19 (22·1)	100 (18·5)	
**Dementia at diagnosis**	6 (7·0)	46 (8·5)	0·63
**BMI at diagnosis** (Kg/m^2)	24·7 [3·7]	24·1 [3·9]	0·18
**ALSFRS-R at diagnosis**	41·8 [5·0]	38·5 (6·9)	**< 0·0001**
**Disease progression rate (points/month) measured at diagnosis**	0·62 [0·65]	1·1 (1·5)	**0·0034**
**FVC at diagnosis, mean [SD]**	86·4 [24·9]	92·9 [23·9]	0·12
**MiToS stage at diagnosis**	0·19 [0·58]	0·32 [0·77]	0·11
**King's stage at diagnosis**	1·55 [0·81]	2·01 [0·98]	**< 0·0001**
**Riluzole treatment**	82 (95·4)	477 (88·2)	0·047

SD: Standard Deviation; UMN-P: Upper Motor Neuron predominant; BMI: Body Mass Index; ALSFRS-R: ALS Functional Rating Scale – Revised.
Based on the nature of the clinical variable under investigation, the homogeneity between cases and controls was assessed by Student's T test or chi-square test. The resulting p-values are representative of good homogeneity of clinical features among tested groups.

Supplementary Table 2: Standardized mean differences after propensity score matching. Demographic and clinical characteristics of TUDCA-treated and non-TUDCA-treated patients with ALS after propensity score matching are reported in Table 1.

Clinical features	SMD
Sex, male	0·040
Months from onset to diagnosis, mean	0·036
Age at onset, mean	0·005
Phenotype	0·025
BMI at diagnosis, mean	0·071
ALSFRS-r at diagnosis, mean	0·022
Disease progression rate at diagnosis, mean	0·035
FVC at diagnosis, mean	0·025

BMI: Body Mass Index; ALSFRS-R: ALS Functional Rating Scale – Revised. FVC: Forced Vital Capacity.

Supplementary Table 3: Relevance of covariates included in Cox regression analyses as confounding factors on survival time.

Covariates	Hazard Ratio
	p-value
**Survival Analyses (from onset to death/tracheotomy) performed on PSM cohort**	
Level I: Survival: from onset to death/tracheotomy (treatment impact)	
• Patients treated with riluzole (n=234)	0·99
• Delay of TUDCA initiation from onset (n=86)	**0·0013**
• Delay of TUDCA initiation from diagnosis (n=86)	0·55
• FVC value at the baseline (n=125)	**0·0073**
Level II: Survival: from onset to death/tracheotomy (duration impact)	
• Patients treated with riluzole (n=234)	0·99
• Delay of TUDCA initiation from onset (n=86)	**< 0·0001**
• Delay of TUDCA initiation from diagnosis (n=86)	0·69
• FVC value at the baseline (n=125)	0·022
Level III: Survival: from onset to death/tracheotomy (dosage impact)	
• Patients treated with riluzole (n=234)	0·99
• Delay of TUDCA initiation from onset (n=86)	**0.0022**
• Delay of TUDCA initiation from diagnosis (n=86)	0.56
• FVC value at the baseline (n=125)	**0.0092**
**Analyses performed on sub PSM cohort (excluding TUDCA < 1000 mg/day and their matched controls)**	
**Survival: from onset to death/tracheotomy (treatment impact)**	
• Patients treated with riluzole (n=116)	0·99
• Delay of TUDCA initiation from onset (n=66)	**0·0062**
• Delay of TUDCA initiation from diagnosis (n=66)	0·22
• FVC[a] value at the baseline (n=64)	**0·015**

FVC: Forced Vital Capacity. The reported p-values estimate the relationship between selected covariates and survival probability. They were determined by using the same Cox regression model applied for estimating the Hazard Ratios of TUDCA treatment, duration and doses reported in Table 3.

Supplementary Table 4: Median survival and hazard ratio on patients' subgroups comparing high-dose TUDCA-treated patients and their propensity score-matched non-TUDCA-treated patients

**Subgroups of patients**	**Survival**	**Hazard ratio**		
	Median (CI)	*HR*	*95% CI*	*p-value*
Spinal patients not treated with TUDCA (n=103)	41·5 (33·1-50·5)	-	-	-
Spinal patients treated with TUDCA ≥1000 mg/day (n=54)	93·5 (43·1- 96·1)	0·44	0·26-0·76	**0·0029**
Bulbar patients not treated with TUDCA (n=29)	34·1 (22·4-44·2)	-	-	-
Bulbar patients treated with TUDCA ≥1000 mg/day (n=12)	31·8 (24·5- NA*)	0·81	0·26-2·49	0·71
Familial patients not treated with TUDCA (n=13)	30·5 (10·8- NA*)	-	-	-
Familial patients treated with TUDCA ≥1000 mg/day (n=7)	56·2 (20·3- NA*)	0·52	0·11-2·54)	0·42
C9ORF72 patients not treated with TUDCA (n=7)	11·4 (6·5-16·5)	-	-	-
C9ORF72 patients treated with TUDCA ≥1000 mg/day (n=5)	- (20·3- NA*)	0·13	0·15-1·16	0·068
Slow progressors not treated with TUDCA (n=50)	57·34 (41·6 - NA*)			
Slow progressors treated with ≥1000 mg/day (n=25)	96·2 (93·5-NA*)	0·17	0·05-0·60	**0·0065**
Intermediate progressors not treated with TUDCA (n=49)	41·6 (33·1-54·6)			
Intermediate progressors treated with ≥1000 mg/day (n=33)	43·0 (33·1-56·2)	0·90	0·45-1·76	0·75
Fast progressors not treated with TUDCA (n=33)	16·5 (13·0-19·9)			
Fast progressors treated with ≥1000 mg/day (n=8)	20·4 (3·9-NA*)	0·84	0·32-2·20	0·73
Intermediate and fast progressors not treated with TUDCA (n=82)	31·6 (23·8-38·6)			
Intermediate and fast progressors treated with ≥1000 mg/day (n=41)	41·7 (31·1-56·2)	0·58	0·34-0·98	**0·043**

NA: not available. CI: confidence interval. HR: Hazard Ratio. * the CI cannot be estimated because a low observations' size or a higher number of censored observations. Median survival times from Kaplan-Meier analyses and Hazard Ratio descriptors from univariable Cox regression analyses were reported, respectively. For analyses on progressor subgroups, the obtained results should be considered as suggestive of possible trends, due to possible biases because of the inhomogeneity of case/control groups due to their limited sample size as demonstrated by the Breslow-Day Test ($p<0.01$).

APPENDIX

ERRALS study group:
Project coordinator: Prof. J. Mandrioli
Collaborating centers:
Department of Neurosciences, Azienda Ospedaliero Universitaria di Modena and Department of Biomedical, Metabolic and Neural Sciences, University of Modena and Reggio Emilia, Modena, Italy (Jessica Mandrioli, Nicola Fini, Ilaria Martinelli, Elisabetta Zucchi, Giulia Gianferrari, Laura Ferri, Cecilia Simonini, Annalisa Gessani, Maria Caputo, Andrea Ghezzi, and Marco Vinceti);
Dipartimento di Scienze Biomediche e Neuromotorie, University of Bologna, and IRCCS Istituto delle Scienze Neurologiche di Bologna, Bellaria Hospital, Bologna, Italy (Veria Vacchiano and Rocco Liguori);
IRCCS Istituto delle Scienze Neurologiche di Bologna, Bellaria Hospital, Bologna, Italy (Fabrizio Salvi, Ilaria Bartolomei and Roberto Michelucci);
Dipartimento di Scienze Biomediche e Neuromotorie, University of Bologna, Bologna (Pietro Cortelli);
IRCCS Istituto delle Scienze Neurologiche di Bologna, Department of Neurology and Stroke Center, Maggiore Hospital, Bologna, Italy (Anna Maria Borghi, Andrea Zini)
IRCCS Istituto delle Scienze Neurologiche di Bologna, UOC Interaziendale Clinica Neurologica Metropolitana (NeuroMet), Bologna, Italy (Rita Rinaldi and Pietro Cortelli);
Department of Neurosciences and Rehabilitation, St Anna Hospital, Ferrara (Elisabetta Sette, Valeria Tugnoli);
Department of Neuroscience, University of Ferrara, Ferrara (Maura Pugliatti);
Department of Neurology, IRCCS Arcispedale Santa Maria Nuova, Reggio Emilia (Elena Canali, Luca Codeluppi and Franco Valzania);
Department of General and Specialized Medicine, University Hospital of Parma, Parma, Italy (Lucia Zinno, Filippo Stragliati, Pietro Anceschi, Andi Nuredini, Sonia Romano, Alessandro D'Orsi, and Liborio Parrino);
Department of Neurology, Fidenza Hospital, Parma, Italy (Doriana Medici and Giovanna Pilurzi);
Department of Neurology, G. Da Saliceto Hospital, Piacenza (Emilio Terlizzi, Donata Guidetti);
Department of Neurology, Carpi Hospital, Modena (Silvia De Pasqua, Mario Santangelo);
Department of Neurology, Imola Hospital, Bologna (Paola De Massis);
Department of Neurology, Faenza and Ravenna Hospital, Ravenna (Matteo Gizzi, Mario Casmiro, Pietro Querzani);
Department of Neurology, Bufalini Hospital, Cesena, Italy (Simonetta Morresi, Maria Vitiello, and Marco Longoni);
Department of Neurology, Forlì Hospital, Forlì, Italy (Alberto Patuelli, Susanna Malagù, Francesca Bianchi, and Marco Longoni);
Department of Neurology, Infermi Hospital, Rimini, Italy (Marco Currò Dossi, Cristiana Ganino, and Marco Longoni);
Department of Hospital Services, Emilia Romagna Regional Health Authority, Bologna (Salvatore Ferro).
CREAGEN Research Center for Environmental, Genetic and Nutritional Epidemiology, University of Modena and Reggio Emilia, Modena, Italy (Marco Vinceti)
