# Supplementary material for: Effect of tauroursodeoxycholic acid on survival and safety in amyotrophic lateral sclerosis: a retrospective population-based cohort study
Source: eClinicalMedicine. 2023 Oct 5;65:102256. doi: 10.1016/j.eclinm.2023.102256 (PMC10570688; doi:10.1016/j.eclinm.2023.102256)
Supplement: ERRALS group for PUBMED [file mmc2.docx]

**ERRALS members to be listed as collaborators in PubMed**

| **First and middle Name** | **Surname** |
| --- | --- |
| Laura | Ferri |
| Annalisa | Gessani |
| Rocco | Liguori |
| Pietro | Cortelli |
| Roberto | Michelucci |
| Fabrizio | Salvi |
| Ilaria | Bartolomei |
| Anna Maria | Borghi |
| Andrea | Zini |
| Rita | Rinaldi |
| Valeria | Tugnoli |
| Maura | Pugliatti |
| Luca | Codeluppi |
| Franco | Valzania |
| Filippo | Stragliati |
| Andi | Nuredini |
| Sonia | Romano |
| Alessandro | D’Orsi |
| Liborio | Parrino |
| Doriana | Medici |
| Giovanna | Pilurzi |
| Emilio | Terlizzi |
| Donata | Guidetti |
| Silvia | De Pasqua |
| Mario | Santangelo |
| Paola | De Massis |
| Matteo | Gizzi |
| Mario | Casmiro |
| Pietro | Querzani |
| Simonetta | Morresi |
| Maria | Vitiello |
| Marco | Longoni |
| Alberto | Patuelli |
| Susanna | Malagù |
| Francesca | Bianchi |
| Marco | Currò Dossi |
| Cristiana | Ganino |
