# Supplementary material for: Effect of tauroursodeoxycholic acid on survival and safety in amyotrophic lateral sclerosis: a retrospective population-based cohort study
Source: eClinicalMedicine. 2023 Oct 5;65:102256. doi: 10.1016/j.eclinm.2023.102256 (PMC10570688; doi:10.1016/j.eclinm.2023.102256)
Supplement: Italian abstract [file mmc3.docx]

***The following translations in Italian were submitted by the authors and we reproduce them as supplied. They have not been peer reviewed. Our editorial processes have only been applied to the original abstract in English, which should serve as reference for this manuscript.***

**Riassunto**

**Background:** L'acido tauroursodeossicolico in formulazione orale (TUDCA) è un farmaco disponibile in commercio e attualmente testato in alcune sperimentazioni in pazienti affetti da sclerosi laterale amiotrofica (SLA) sia da solo che in combinazione con il fenilbutirrato di sodio. Questo studio retrospettivo ha lo scopo di esplorare in un contesto del mondo reale se il TUDCA abbia avuto un effetto sulla sopravvivenza nei pazienti con SLA trattati con questo farmaco rispetto a coloro che hanno ricevuto esclusivamente i trattamenti standard.

**Metodi:** il nostro studio è stato condotto in Emilia Romagna (Italia), attingendo al registro regionale della SLA attivo dal 2009 ed utilizzando il metodo del “propensity score”. Dei 627 pazienti con SLA diagnosticati ed inseriti nel registro dal 1° gennaio 2015 al 30 giugno 2021 con informazioni disponibili sulla data di morte e/o tracheostomia, 86 pazienti hanno assunto il TUDCA e sono stati abbinati in un rapporto di 1:2 con pazienti che hanno ricevuto esclusivamente i trattamenti standard, in base all'età di insorgenza, al sesso, al fenotipo, all’intervallo diagnostico, all'indice di massa corporea alla diagnosi, e al punteggio ottenuto alla versione rivista della scala di valutazione funzionale della SLA (ALSFRS-R) alla prima visita e al tasso di progressione della malattia alla prima visita. L'outcome primario è stato considerato la differenza di sopravvivenza (tempo dall'inizio dei sintomi alla tracheostomia e/o morte) tra i pazienti esposti al TUDCA e quelli non esposti.

**Risultati**: In totale 86 pazienti trattati con TUDCA sono stati abbinati a 172 pazienti con SLA non trattati. I pazienti esposti al TUDCA sono stati stratificati in base al dosaggio (inferiore e uguale o superiore a 1000 mg/giorno) e alla durata (inferiore e uguale o superiore a 12 mesi) del trattamento. La sopravvivenza complessiva mediana è stata di 49,6 mesi (IC al 95% 41,7–93,5) tra i pazienti trattati con TUDCA e di 36,2 mesi (IC al 95% 32,7–41,6) nei controlli, con una riduzione del rischio di morte per i pazienti esposti a dosaggi più elevati di TUDCA (HR 0,56; IC al 95% 0,38-0,83; p=0,004) rispetto ai controlli e ai pazienti con dosaggi più bassi di TUDCA. Il TUDCA è stato ben tollerato, ad eccezione di una minoranza di pazienti (n=7; 8,1%) che hanno interrotto il trattamento a causa di effetti collaterali, principalmente gastrointestinali e di lieve entità; solo 2 eventi avversi hanno richiesto l'accesso in ospedale ma si sono risolti senza esiti.

**Interpretazione:** Questo studio esplorativo condotto su un registro di popolazione, mostra che i pazienti con SLA trattati con TUDCA potrebbero avere una sopravvivenza prolungata rispetto ai pazienti che ricevono esclusivamente i trattamenti standard. Ulteriori studi prospettici randomizzati sono tuttavia necessari per confermare l'efficacia e la sicurezza di questo farmaco.

**Finanziamento** Regione Emilia Romagna.
